# Supplementary material for: Evaluation of the 50% Infectious Dose of Human Norovirus Cin-2 in Gnotobiotic Pigs: A Comparison of Classical and Contemporary Methods for Endpoint Estimation
Source: Viruses. 2020 Aug 28;12(9):955. doi: 10.3390/v12090955 (PMC7552045; doi:10.3390/v12090955)
Supplement: Supplementary file 1 [file viruses-12-00955-s001.pdf]

Supplementary Material

Infostat connection to R

https://www.infostat.com.ar

Model specification > General linear mixed model

Dependent variable = response, can be:

PT = peak titer of virus shed

ONSETS= days to onset of virus shedding

SDUR = duration of virus shedding

LogAUC= log10 of the area under the curve of virus shedding

DOSE group = fix variable

Gn.pig in each dose group = random variable

Variance-covariance matrix= varIdent

```
mlm.model.001_PT_REML<-lme(response~1+DOSE

,random=list(Gn.pig=pdIdent(~1))

,weights=varComb(varIdent(form=~1|DOSE))

,method=\"REML\"

,control=lmeControl(niterEM=150

,msMaxIter=200)

,na.action=na.omit

,data=mlm.modeloR.data04

,keep.data=FALSE)
```

##### ANOVA Ramesh, 2020. R SCRIPT ###############

##### Package needed ######

```
install.packages("car")

install.packages("broom")

install.packages("gplots")

install.packages("fBasics")

install.packages("agricolae")

install.packages("nlme")

install.packages("lsmeans")

install.packages("ggplot2")
```

```
#### Package activation #######

library(broom)

library(car)

library(gplots)

library(fBasics)

library(agricolae)

library(nlme)

library(lsmeans)

library(ggplot2)

library(lme4)

library(RcmdrMisc)

library(lattice)

library(multcomp)

##### Call excel file with the dataset #####

ID50NOV

names(ID50NOV)

ID50NOV$"Dose ID"<-as.factor(ID50NOV$"Dose ID")

ID50NOV$TRAT<-as.factor(ID50NOV$TRAT)

summary(ID50NOV)

##### Exploring the data ######

Response = ONSETS, SDUR, LogAUC, PT, ONSETD, AUCD, DDUR, CUMDSCORE

boxplot(response~LogDOSE, data=ID50NOV,xlab="Norovirus challenge dose",

        ylab="Response=AUC virus shedding", col="red", digits=2)

# scatter plot

plot(response ~ LogDOSE, ID50NOV, ccol="red", pch=20, ci.label=TRUE,digits=2, ylim=c(0,6), cex=2)

######## One Way ANOVA ########################

# response = ONSETD, DDUR, CUMDSCORE, AUCD

anovaresponse<-aov(ID50NOV$Sresponse~ID50NOV$TRAT)

summary(anovaresponse)

#control of assumptions

shapiroTest(residuals(anovaresponse))

leveneTest(ID50NOV$response,ID50NOV$TRAT)
```

```
bartlett.test(response~TRAT)
```

#post ANOVA multiple comparison

```
TukeyHSD(anovaresponse)

pairwise.t.test(ID50NOV$response,ID50NOV$TRAT,p.adjust.method="bonferroni")

(test.response<-lsmeans(anovaresponse, pairwise ~ TRAT))

plot(test.response$contrasts)

library(agricolae)
```

#MODIFIED TUKEY TEST

```
comparison <- HSD.test(modelo2,"interaction", group=TRUE,
                        main="interaction effect"))

bar.group(comparison$groups,ylim=c(0,45),density=4,border="blue")
```
